# Participatory Democracy, Community Organizing and the Community Assessment of Freeway Exposure and Health (CAFEH) Partnership

**DOI:** 10.3390/ijerph14020149

**Published:** 2017-02-04

**Authors:** Linda Sprague Martinez, Ellin Reisner, Maria Campbell, Doug Brugge

**Affiliations:** 1 Macro Department, Boston University School of Social Work, 264 Bay State Road, Boston, MA 02215, USA; 2 Somerville Transportation Equity Partnership, 51 Mt. Vernon Street, Somerville, MA 02145, USA; Reisnere51@gmail.com; 3 Department of Public Health and Community Medicine, Tufts University School of Medicine, 136 Harrison Avenue, Boston, MA 02111, USA; Maria.Campbell@tufts.edu (M.C.); doug.brugge@gmail.com (D.B.)

**Keywords:** community-based participatory research (CBPR), community–academic partnerships, team science, near highway exposure

## Abstract

*Background:* Conflicting interests, power imbalance and relationships characterized by distrust are just a few of the many challenges community–academic research partnerships face. In addition, the time it takes to build relationships is often overlooked, which further complicates matters and can leave well-intentioned individuals re-creating oppressive conditions through inauthentic partnerships. This paper presents a novel approach of using meeting minutes to explore partnership dynamics. The Community Assessment of Freeway Exposure and Health (CAFEH) partnership is used as an illustrative case study to identify how community academic partnerships overcome the challenges associated with community-based participatory research (CBPR). CAFEH is a study of ultrafine particle exposure (UFP) near highways in the Boston, MA area. *Methods:* Qualitative analysis was applied to meeting minutes and process evaluation reports from the first three years of the CAFEH study (*n* = 73 files). In addition, a group meeting was held with project partners in order to contextualize the findings from the document analysis. *Results:* The three most commonly referenced challenges included language barriers, the overall project structure and budgetary constraints. Meanwhile, a heavy emphasis on process and an approach steeped in participatory democracy facilitated CAFEH’s ability to overcome these challenges, as well as sustain and augment strong partnership ties. *Conclusions:* This experience suggests that leadership that incorporates an organizing approach and a transformational style facilitates CBPR processes and helps teams surmount challenges.

## 1. Introduction

In memory of Cheri Lieberman, Ph.D. who evaluated and informed our partnership processes.

Improving environmental health and community wellbeing requires the coordination of multiple stakeholders working in concert to address complex problems. However, assembling interdisciplinary teams that span the academy and the community can pose real challenges for both community partners and university stakeholders, despite the many benefits of participatory research partnerships. We examined the Community Assessment of Freeway Exposure and Health (CAFEH) as a case study. CAFEH is a community academic research partnership that studies health effects of and remedies to exposure to ultrafine particle exposure (UFP) pollution near highways.

The benefits of community-based participatory research (CBPR) can be many. Engaging communities can increase a study’s sustainability and efficacy, while addressing community specific priorities [1]. CBPR can also facilitate resource sharing and increase local capacity and participation [2]; and while the direct effects of participatory research on health are limited, positive correlations between this approach and improvements in health have been noted [3]. CBPR comes with challenges as well as benefits. Conflicting interests can result in partner disagreements [4]; similarly differences in language use among partners can generate conflict [5]. Meanwhile, power dynamics among partners can have a crippling effect on communication and trust [6]. Furthermore, CBPR is time intensive [7] as building trust relationships requires both intentionality and transparency in addition to time [8].

Understanding the challenges associated with CBPR is just as important as understanding its benefits if the science of CBPR is to be advanced. Studying partnership processes allows CBPR researchers to advance the field by uncovering barriers to partnership and strategies used by community and academic stakeholders to overcome them. Systematically examining partnership processes can inform the work of the partnership as well as the field more broadly, by identifying factors that facilitate trust, mutual respect, communication, equity and participatory democracy.

The CAFEH partnership was established nearly a decade ago. Since its inception, the partnership has grown significantly in both size and scope. Its complexity and evolution over time makes CAFEH an interesting case study for those interested in the processes associated with research partnerships. The goal of the inquiry described in this paper was to identify factors that have helped CAFEH to surmount challenges associated with CBPR. As such, the primary research question was, what challenges did CAFEH experience in the early years of the partnership? What strategies did they employ to overcome challenges associated with CBPR processes? In short, what makes CAFEH work? In order to explore these questions, meeting minutes from the first three years of the partnership were analyzed, as were process evaluation reports. In addition, a group meeting was held with project partners in order to contextualize themes that emerged during the document review.

This paper adds to the literature by presenting an illustrative case study. All too often the successful outcomes of partnerships overshadow the challenges. We highlight here the factors that facilitated CAFEH’s ability overcome challenges as well as sustain and augment strong partnership ties. A brief literature on the barriers to CBPR is followed by a description of the CAFEH study and an overview of the research methods. The findings are then discussed.

### 1.1. Background

The last decade has seen an increase in CBPR funding. With this increase, more researchers are reporting on CBPR studies, both outcomes and processes. Numerous challenges associated with CBPR have been reported in the literature [1,9,10,11]. These challenges range from distrust and competing interests to the role of race, power and privilege [10,12,13]. In addition, the time associated with CBPR poses a challenge, causing some researchers to shy away from partnership approaches [12].

Mutual trust is a key tenet of CBPR, however building trust relationships is more easily said than done. This is particularly true given that the divide between academic institutions and the communities within which they sit is real [12]. The reputation of the university in the community can inhibit the ability of even well-intentioned researchers to establish trust relationships with community stakeholders. Factors that may serve as a point of contention for community members may include: a history of students and faculty dropping in, collecting information and leaving a history of redevelopment viewed unfavorably by the community; and the inequitable distribution of resources whereby the university is infrastructure-rich at the expense of the communities [14,15,16,17,18].

Just as challenges establishing trust may arise as a result of policies and past relationships beyond the control of the researcher, social and racial location are important considerations in CBPR. Chavez et al. (2008) discuss the role of race, ethnicity, power and privilege in CBPR, describing specifically how power imbalances and systems of oppression can unknowingly impede the development of trust relationships and the partnership process [13]. Similarly, Travers et al. (2013) discusses the inherent power imbalances between community members and researchers, as well as the ways in which community level trauma, similarly to internalized racism can create tension within oppressed groups [11].

Communication barriers associated with language use also pose a challenge to CBPR partnerships [5]. Power is embedded within language [19] and language that is overly technical can serve to disempower communities and threaten trust [11]. Moreover, communication barriers can get in the way of partnerships and detract from the work [3,20]. Developing a shared vision and maintaining trust calls for transparency and intentionality [9]. This requires spending time on the process, and being upfront from the start about everything from the finances to preconceived notions and expectations [9]. Chavez and colleagues (2008) [13] provide a powerful metaphor describing CBPR as a dance, whereby new partners need to practice, all the while listening, observing, moving forward and stepping back, as they learn from one another until they are eventually able to move together in unison. The CAFEH partnership today moves in relative unison, but was it always like that? What were the challenges they faced as they came together and how were they able to overcome them? In the section that follows the CAFEH partnership is described in detail.

### 1.2. CAFEH Overview

Studies have shown that living near major roads and highways is linked to an increased risk in many chronic diseases. This was concerning community activists in Somerville, MA given the community’s proximity to a major highway. Over a decade ago, they brought their concern to researchers at Tufts University. The initial idea was to have one of the university researchers serve as a technical consultant to a lawsuit they were considering. As the lawsuit never developed, the Somerville partners brought a new idea to the table, to write a research proposal to further document and understand the issue of traffic pollution and its impact on health. Additional stakeholders were identified and invitations to participate were extended to both community and university researchers. The team met over a period of months to prepare the research grant for submission to the National Institute of Health (NIH). Following a first round of reviews that resulted in a good score but no funding, the proposal was revised and resubmitted. The process from the start of writing to funding took nearly two years.

In 2008, the CAFEH partnership was awarded a Community-Based Participatory Research grant (R01) from the National Institute of Environmental Health Sciences (NIEHS) to test associations between exposure to UFPs near highways and cardiovascular disease risk in older adults. The initial CAFEH team included academic researchers from engineering and public health at Tufts and Harvard Universities, together with a community partner from the City of Somerville, Boston Chinatown and Boston public housing.

Over the course of the grant the CAFEH partnership collected extensive field data both through mobile air pollution monitoring and by recruiting about 700 participants. The study design and findings have been reported elsewhere and are important to the process analysis presented in this paper only as background and context [21,22,23]. Briefly, the study collected data in three sets of paired neighborhoods over the course of three years. The air pollution data was used to model UFP levels in each neighborhood at a fine grain (hourly for a year at 20 m). The predicted UFP levels were adjusted for participant time activity patterns and individualized exposures were assigned. These exposures were found to be associated with blood biomarkers of inflammation, suggesting that UFP exposure might increase risk for cardiovascular disease. The results were the first to show the association of near highway UFP exposure with indicators of health.

### 1.3. Sustaining Momentum

**Figure 1:**
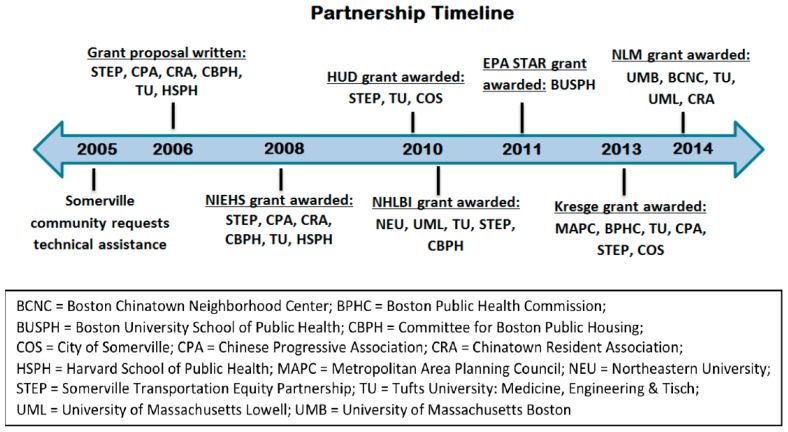
The Community Assessment of Freeway Exposure and Health (CAFEH) Partnership Timeline.

In order to address new and remaining concerns, the CAFEH partnership sought funding to conduct longitudinal research on UFP exposure, to test in-home air filtration interventions and to explore implementation of community tactics through policy and practice, resulting in additional grants and substantial expansion beyond the research envisioned in the original grant. Figure 1 illustrates the expansion of the partnership over time as new funding was awarded. Additional funding included fellowships for two PhD students from the Environmental Protection Agency, a grant from Housing and Urban Development to test air filtration in homes of public housing residents living near a highway, a collaboration with the Boston Puerto Rican Health Study on their center grant that added traffic pollution to the health factors assessed in their cohort. Funding was also received from the Kresge Foundation to work on translating the research into policy and practice at the community level and from the National Library of Medicine to conduct environmental education in Boston Chinatown. In 2016, CAFEH received another R01 from NIEHS to conduct a community-level intervention to address traffic-related pollution in Boston Chinatown and Somerville.

CAFEH began and has remained true to its community-based participatory research model. This has meant that the work is led by a steering committee, which remains intact today, which includes representation from university-based researchers, community partners, students, field staff and consultants. The steering committee makes strategic level decisions while the details of each project are addressed in more focused subcommittees, which were added during the initial years of the project, and also have broad representation. Community participation in what is often technical science has been extensive and community partners are co-authors of many academic publications, including papers in high-end environmental science and environmental epidemiology journals.

### 1.4. CAFEH’s Impact

CAFEH has sought to be and achieved to be productive on both academic levels and in ways that translate research into a beneficial impact on the partner communities. More than 30 academic papers have been published so far, with another 15–20 expected from the funding through 2016. Papers have been published in multiple areas of scholarships, including environmental epidemiology, environmental science, exposure science, social science, and community-engagement. Numerous articles are in journals with high impact factors such as *Environmental Health Perspectives, Environment International, Environmental Science & Technology* and the *Journal of Exposure Science and Environmental Epidemiology*.

The Kresge-funded work led to engagement with and influence on several developers of near highway housing and a school project. These interactions led to design changes in several developments as well as relationships with designers, architects and planners that continue to provide opportunities for engagement. The project also developed a draft zoning ordinance for the City of Somerville that is under consideration for inclusion in the city’s new zoning code [24]. Overall, the relationship between CAFEH and the City of Somerville has been strong, with recent requests from the city to conduct air pollution monitoring at high traffic sites to inform future development on those sites.

In sum, the CAFEH team has had a successful track record with meaningful accomplishments and partner participation has remained strong. This paper specifically explores the early stages of the CAFEH partnership, in an attempt to identify factors that have led to their sustainability over the years, and how they have been able to overcome the many challenges they faced early on.

## 2. Methods

In order to better understand partnership processes associated with CAFEH and to identify strategies employed to overcome CBPR related challenges, we examined documents from the first three years of the partnership, 2008–2011. During the early stages when partnerships are forming, processes related to roles, responsibilities, decision-making and communication, are generally established and put into place. We therefore examined meeting minutes from the first three years of the study. In addition, we explored yearly reports prepared for CAFEH by an external process evaluator that was required by the funder. After the initial document review key themes were discussed with project partners in order elicit: (1) more details about the context in which decision-making occurred; (2) partner perceptions of the identified themes; and (3) additional themes of importance to CAFEH partners.

### Document Review

The document review was focused on two sources, steering committee meeting minutes (*n* = 70) and evaluation reports (*n* = 3). Steering committee minutes were succinct and did not include details of who said what or the complete flow of conversations. Instead they tended to summarize main outcomes of discussions. Process evaluation reports were based on surveys and key informant interviews with steering committee members and included narrative interpretation with selected quotes. Items explored in evaluation reports included: clarity of the project focus and role; perception of steering committee composition; participation and attendance; meeting strengths, weaknesses and pace; perceptions of conflict resolution strategies and decision-making; how meeting information is conveyed/disseminated to those not in attendance; workload between meetings; satisfaction with the meetings and the decision-making process; and insider/outsider dynamics. Respondents were also provided with the opportunity to discuss additional topics not included.

All word files with steering committee minutes and process evaluation reports were converted to text files. Converted files were then uploaded to N6 QSR, data qualitative data management package [25]. Transcripts were generated and a sample was coded by two researchers. Systematic content analysis that involved the identification, labeling and categorization of the data was conducted. The researchers then met to compare categories and to identify key themes. Using the themes identified, a codebook was developed which was then applied to the remaining interviews. The main themes identified were related to strengths and weaknesses associated with: (1) the steering committee; (2) project logistics; (3) budgetary constraints; and (4) community outreach.

Reports for each of the codes were run and a summary was prepared and reviewed with the study principle investigator. Based on the key themes in the report, discussion group questions were developed. Questions explored groups’ perceptions of the challenges related to the steering committee process, budgetary constraints and logistics identified in the minutes and how CAFEH was able to overcome them. Investigators and staff were invited to participate in a group discussion, the goal of which was to elicit additional information related to problem solving and factors that may have helped to contribute to CAFEH’s success. During the group session (*n* = 4), partners were asked to outline successes and challenges associated with the project. Themes from the discussion were recorded by hand and then coded thematically by two independent researchers.

## 3. Results

The analysis of meeting minutes and reports uncovered a number of challenges experienced by CAFEH partners during their initial years. As indicated in the methods, overall themes identified were related to strengths and weaknesses associated with: (1) the steering committee; (2) project logistics; (3) budgetary constraints; and (4) community outreach. The three most commonly referenced challenges were language barriers, the overall project structure and budgetary constraints. In addition to challenges identified, the documents reviewed contained some solutions for addressing each of the challenges. The discussion with the partner group provided additional context about what facilitated their ability to overcome challenging circumstances.

### 3.1. Language Barriers

Language barriers were the most commonly discussed challenges for the CAFEH partnership. Two distinct but interrelated sets of barriers emerged with respect to language. First, the study was being carried out in diverse immigrant communities and project staff hired from communities had varying degrees of English fluency. Although an asset to the partnership overall, linguistic diversity posed some challenges at meetings and with protocols. Meeting notes and evaluation reports described the need to translate process evaluation protocols and materials for Chinatown partners, as well as concerns about the clarity with which information was presented during meetings. Similarly, a process evaluation interviewee shared, “of concern are LEP (limited English proficiency) folks, assuring they understand. They don’t ask a lot of questions and are quiet at meetings. It may be language”. It was further reported that having an interpreter available diminished this concern. “We have an interpreter now and things are getting better”.

However, language barriers went beyond the need for interpreter services. Meeting notes revealed concern over scientific language or lexicon used during discussions. This was generally referred to as “heavy science” or “technical stuff”. Here language barriers were seen as resulting from both the interdisciplinary nature of the team and varying abilities of steering committee members to digest scientific content. During a process evaluation interview conducted in 2008 one partner shared, “I glaze over when we do the science stuff”.

The challenge presented by scientific language led CAFEH to shift its overall leadership structure and by 2011, 100% of partners interviewed reported that they almost always understood what was being discussed at meetings. As described by one evaluation participant “A lot of heavy science was presented to us clearly”. This shift was likely related to the shift to a subcommittee structure in which more technical details were addressed in the subcommittees instead of the steering committee. In addition, partners described being sensitive to this barrier, which increasingly led to more intentional language use and avoidance of jargon.

### 3.2. Project Structure and Decision-Making

Project structure initially posed a problem for the group. CBPR calls for collective decision-making and partner involvement in all aspects of the study. Meeting notes indicate that early on CAFEH leaders may have taken this a bit too far. Initial steering committee meetings were long and project partners reported during process evaluation interviews that too much information was being shared in the context of the steering committee. As described by one evaluation interview participant, “sometimes things get bogged on particular topics that could be discussed at smaller meetings, recruitment issues for example”. This sentiment contributed to the decision to shift to the subcommittee structure.

Two subcommittees were formed, one focused on health and the other on environmental science. In addition, fieldwork meetings were set up by the project manager. Subcommittees had both university and community representation and meeting minutes were made available. Decisions that did not impact the overall direction of the project were made in subcommittees and the steering committee became a body focused on strategic decision-making, and a place where committee reports were shared. In addition, meeting facilitation shifted from university-based leadership to shared facilitation with a community investigator. This change was seen by partners as beneficial, “It has improved in the past year. It is good that others are facilitating.”

Partners reported decisions are “discussed until everyone comes to a consensus, important things are voted on, usually the votes are unanimous”. Moreover, it was reported that “there are not many disagreements. Generally, it (a given issue) is discussed at the meeting and if there is a conflict we get input from others at the table. The group works together well”. Table 1 below, Decision-Making, illustrates partner perceptions of how decisions are made.

### 3.3. Budget Constraints

Budgetary constraints emerged as a significant concern dominating the meeting minutes during the project’s second year. Constraints resulted from unanticipated expenses associated with field staff, the high cost of equipment and a $25,000 error in budget figures that led to overspending in the initial project year. Budgetary constraints both limited the number of additional community members that could be added and reduced the number of air pollutants that could be accounted for. Although much time was spent in discussing budget constraints they did not negatively impact the team over the long term, largely because the team decided to reduce the target data collection for the final study area.

Partners reported transparency improved the team’s ability to be flexible when the budget needed to be modified. It helped tremendously that CAFEH had an open budget from the start. Everyone on the steering committee knew how funds were allocated and the amount of subcontracts as well as the amount in allocated on each line of the main budget, this helped to reduce distrust. Moreover, people were willing to reduce their time in order to save the budget because they were committed to the work of the project. In times of budgetary uncertainty circling back to the overall project goals and voicing commitment, together with transparency, boosted morale and facilitated the team’s ability to move through a difficult period.

### 3.4. What Makes CAFEH Work?

During the course of the group meeting, partners were asked to talk about why things work and why they are able to overcome challenges and “work well” together. Responses were related to transparency and an intentional approach to leadership, “Doug does a good job making sure all the issues are out on the table, before a consensus is reached. Conversations are thoughtful”. Others, reported appreciating the democratic process used and that “people almost always felt that their point of view was respected.” One person reported feeling respected “even if I’m wrong”. There was also a recognition by the group that all partners were committed to the research focus. Community members were willing to sit through complex scientific conversations, while those deep in the science were cognizant of the complexity and were careful to ensure people remained engaged. This may have been due to the fact that community members drove the project from inception, having brought it to academic researchers, they felt responsible and were recognized by researchers as catalyzing the project. It was also expressed during the meeting that, “Doug is really good at rounding us up and bringing us together”.

### 3.5. Unexpected CAFEH Outcomes

Community partners involved in the CAFEH study were from very different communities. Somerville partners describe appreciating the opportunity to work with Chinatown partners. Moreover, it was reported that both community partners learned from one another as they both approached organizing differently in their respective communities. In short, new social ties were gained through CAFEH, participation increased partner knowledge as well as organizational ties.

## 4. Discussion

The language barriers discussed by the CAFEH partnership are consistent with the literature. Sprague Martinez and colleagues (2012) found language barriers pose challenges for community academic partnerships, as language can be exclusionary, creating power differentials between partners which is particularly true in situations where discipline specific scientific jargon is used [5]. CAFEH partners addressed language barriers early on and throughout the project which sought to ease power imbalances between partners. However, the dynamics of race and power were different for CAFEH. The Somerville partners were white, highly educated and felt comfortable challenging researchers. In the case of the Chinatown partners, they had a long standing relationship with the study Principal Investigator (PI) so much of what gets in the way of partnership may have already been worked out in partnerships years earlier.

CAFEH partners demonstrated a great deal of flexibility and responsiveness to the needs of the overall group. Flexibility has been identified in the literature as a key tenet of transdisciplinary research [26]. This openness and flexibility created a level of positive energy that helped CAFEH to get through challenges such as language barriers and a heavy time commitment associated with multiple meetings. Similarly, findings indicate that no CAFEH partner is any more or less important. All (students, community leaders, university researchers and staff) were valued and over time they developed a collective CAFEH identity. This holistic approach, also a key tenet of transdisciplinary research [26], may have left partners open to new ideas by providing space for them to hear one another, which also allowed them to think beyond their own disciplines. This may have additionally contributed to their advanced appreciation for the diverse disciplinary perspectives at the table.

In discussions about what worked with CAFEH partners, they often referred back to the team leadership and the way in which leadership was able to organize the group. CAFEH leadership relied on community organizing to bring diverse stakeholders together. Focusing on the issue at hand, near highway exposure to UFP, the team was able to build deeper connections and enhance existing relationships [27]. By meeting team members where they were at and gaining and understanding individual values, leadership was able to establish a greater degree of trust among the group [28].

There is a great deal of synergy between transdisciplinary team science and community organizing. In transdisciplinary team science diverse researchers are issue-focused [26], coalescing to answer complex questions. The same is true in community organizing in which individuals work to come to a shared understanding of an issue, working collectively to bring about positive change [29]. Meanwhile, CBPR and community organizing share a values-base and key tenets such as empowerment, critical consciousness and capacity building, having both evolved from Paulo Freire’s ideas on liberation theology and popular education [30,31].

CAFEH partners placed a great deal of value on participatory democracy, with the expectation of direct and active participation of all team members regardless of job status [32]. This may have been because the study PI had previous experience in organizing when he was younger. Nonetheless, it facilitated the distribution of power among the team and assured all partners had meaningful voices and roles in the work [33]. This approach led to empowerment and ultimately greater efficacy among team members. Similarly, CAFEH leadership focused a great deal of attention on capacity building among partners, taking a transformational leadership approach that sought to inspire leadership participation among the team. Finally, CAFEH leadership attempted to attend to the needs of individuals with respect to opportunities for growth. They took a positive approach, worked to motivate others and promoted critical thinking, all of which are associated with effective transformational leaders [34]. This type of leadership often intersects with community organizing because a good organizer is one that attends to individuals’ self-interests in order to motivate them and promote leadership capacity.

In short, there were many facts that contributed to CAFEH’s ability to overcome barriers often associated with CBPR. Most likely, however, their success was primarily related to the amount of time they dedicated upfront to the process. The team was very intentional about process from the start and used process evaluation data to inform their approach. Moreover, what differs from many CBPR studies, partners in CAFEH approached the researchers, with their priority area of focus and those researchers who joined were similarly invested in the topic from the start. Also, despite budgetary constraints CAFEH had a good deal of funding and built in compensated time to focus on the dynamics of partnership processes.

## 5. Conclusions

Leadership style was essential to the effectiveness of the CAFEH partnership; as was an intentional focus on the process and transparency. Like most CBPR partnerships, CAFEH experienced meaningful challenges and confronted power differentials that threatened their survival. Analysis of CAFEH processes indicates leadership style, transparency and an intentional focus on process was critical to their sustainability and growth. Fidelity to the key tenets of CBPR which are rooted in community organizing and participatory democracy, facilitated the team’s ability to recalibrate in times of uncertainty. Moreover, taking the time to hear and validate all voices big and small, enhanced trust relationships, as did the level of transparency with which leadership operated. The case of CAFEH is one that illustrates the benefits of attention to process, power-sharing, participation and equity in environmental health research.

## Figures and Tables

**Table 1:** Decision-making.

Illustrative Quotes	
How are decisions made?	They are discussed by everyone present and we come to a unanimous decision Generally, we reach consensus.
	For the sake of process, we usually vote.
	Ask the committee members everyone gives their opinion and then there is a vote.
	Generally, they are by consensus and sometimes there is a vote. Recently we decided to have more voting members—student reps, field team reps, this has added to the meetings to make people feel more included. Sometimes we defer to the person to be the most knowledgeable on the subject.
	Talk them through people raise different views discussion of pros and cons then we discuss and reach consensus.
	Democratically, important decisions are voted upon and for small decisions we try to reach consensus.
	Basically consensus, people with the correct knowledge and experience share to help others.
